# Classification of real and pseudo microRNA precursors using local structure-sequence features and support vector machine

**DOI:** 10.1186/1471-2105-6-310

**Published:** 2005-12-29

**Authors:** Chenghai Xue, Fei Li, Tao He, Guo-Ping Liu, Yanda Li, Xuegong Zhang

**Affiliations:** 1 MOE Key Laboratory of Bioinformatics / Department of Automation, Tsinghua University, Beijing 100084, China; 2 Laboratory of Complex Systems and Intelligence Science, Institute of Automation, Chinese Academy of Sciences, Beijing 100080, China; 3 School of Electronics, University of Glamorgan, Pontypridd CF37 1DL, UK

## Abstract

**Background:**

MicroRNAs (miRNAs) are a group of short (~22 nt) non-coding RNAs that play important regulatory roles. MiRNA precursors (pre-miRNAs) are characterized by their hairpin structures. However, a large amount of similar hairpins can be folded in many genomes. Almost all current methods for computational prediction of miRNAs use comparative genomic approaches to identify putative pre-miRNAs from candidate hairpins. *Ab initio *method for distinguishing pre-miRNAs from sequence segments with pre-miRNA-like hairpin structures is lacking. Being able to classify real vs. pseudo pre-miRNAs is important both for understanding of the nature of miRNAs and for developing *ab initio *prediction methods that can discovery new miRNAs without known homology.

**Results:**

A set of novel features of local contiguous structure-sequence information is proposed for distinguishing the hairpins of real pre-miRNAs and pseudo pre-miRNAs. Support vector machine (SVM) is applied on these features to classify real vs. pseudo pre-miRNAs, achieving about 90% accuracy on human data. Remarkably, the SVM classifier built on human data can correctly identify up to 90% of the pre-miRNAs from other species, including plants and virus, without utilizing any comparative genomics information.

**Conclusion:**

The local structure-sequence features reflect discriminative and conserved characteristics of miRNAs, and the successful *ab initio *classification of real and pseudo pre-miRNAs opens a new approach for discovering new miRNAs.

## Background

MicroRNAs (miRNA) are non-coding RNAs about 21–26 nucleotide (nt) in length that can play important roles in gene regulation by targeting mRNAs for cleavage or translational repression [1,2]. According to the current understanding, miRNA is transcribed as long primary miRNA, which is processed into 60~70 nt miRNA precursor (pre-miRNA) by nuclear RNase III Drosha [3,4]. The pre-miRNA is transported from nuclear to cytoplasm by Exportin-5 [5,6] and then cleaved into ~22 nt duplexes [2]. Almost all pre-miRNAs have the characteristic of stem-loop hairpin structures. During the biogenesis procedure of a mature miRNA, the hairpin structure of pre-miRNA acts as not only the structure motif for Exportin-5 in nuclear-cytoplasm transportation, but also a substrate for Dicer enzyme [5-7]. This indicates the importance of the secondary structures in the miRNA biogenesis procedure.

Due to the difficulty of systematically detecting miRNAs from a genome by existing experiment techniques, computational methods play important roles in the identification of new miRNAs. As a characteristic secondary structure, the hairpin of pre-miRNA is an important feature used in the computational identification of miRNAs. For examples, MiRscan relies on the observation that the known miRNAs are derived from phylogenetically conserved stem-loop precursor RNAs with characteristic features [8,9]. It successfully predicted hundreds of miRNAs in nematodes and human with a sensitivity of 0.74. The miRseeker [10] was developed for predicting miRNA genes in insects, whereas MIRcheck [11] and MIRFINDER [12] were applied in plants. Recently, the miRAlign [13] aligns the secondary structure of pre-miRNAs to detect miRNAs. However, there can be many sequence segments in a genome that may fold into the similar stem-loop hairpin structures, e.g., about ~11 million hairpins can be folded in the human genome [14], and some 44,000 hairpin candidates can be obtained in *C. elegans*, corresponding to ~4% of the worm genome [15]. Therefore, all those existing methods utilize comparative genomics information besides structure features to predict new miRNAs. A typical idea is to use comparative genomics to filter most of hairpins that are not conserved in related species. Such filtering steps make the methods unable to identify new miRNAs for which there are no known close homologies either due to the limitation of current data or due to the possibly rapid evolution of miRNAs. A latest report shows that the number of non-conserved miRNAs which are missed by the comparative genomics strategy is still large [14]. Furthermore, for a species that does not have a closely related species sequenced, its miRNAs cannot be studied with comparative genomics approaches either.

In this study, we focus on the *ab initio *classification of real pre-miRNA from other hairpin sequences with similar stem-loop features (we call them as pseudo pre-miRNAs). A set of novel features that combines the local continuous structure and sequence information of the stem-loops are proposed. The machine learning method SVM or support vector machine is used to classify two classes based on the features. SVM has been widely applied to the prediction and classification of important biology signals such as promoters [16], translation initiation sites [17], splicing sites [18] and proteins [19]. Recently, SVM was successfully applied to predict new virus miRNAs [20] and functional siRNAs [21]. With the local structure-sequence features we extracted, SVM achieves the accuracy about 90% for distinguishing real vs. pseudo human pre-miRNAs. Interestingly, the SVM classifier trained on human miRNA data can also identify miRNAs of other species across animals, plants and virus with high accuracy, which indicates that the features may reflect a characteristic that is consistent across all species. The classifier is also validated on the latest human miRNA data which were missed by all existing prediction methods [14] and a high accuracy (92.3%) is achieved.

## Results and Discussion

### Human miRNA precursor and pseudo miRNA datasets

Sets of human pre-miRNAs and pseudo-miRNA hairpins are collected to train SVMs and to evaluate the classification performance.

#### Human miRNA precursors

The sequences of human pre-miRNAs are downloaded from the miRNA registry database [22-24] in Sept., 2004 (release 5.0), which contains 207 reported pre-miRNA entries from *Homo sapiens*. Only the pre-miRNAs whose secondary structures do not contain multiple loops are considered, which gives us 193 pre-miRNAs, covering more than 93% of all the reported human pre-miRNAs.

#### Pseudo and candidate miRNA hairpins

Two datasets of pre-miRNA-like hairpins are built. They are sequence segments that have similar stem-loop structures as genuine pre-miRNAs but have not been reported as pre-miRNAs. For the convenience of discussion, we call them as the "CODING" and the "CONSERVED-HAIRPIN" datasets according to the ways we collect them.

The CODING dataset is collected from the protein coding regions. The protein coding sequences (CDSs) of human RefSeq genes with no known alternative splice events are collected. The CDS sequences are extracted according to the UCSC refGene annotation tables [25,26]. We join the CDS sequences together and extract non-overlapping segments from it, keeping the length distribution of the extracted segments identical with that of human pre-miRNAs. The secondary structures of the extracted segments are predicted using RNAfold [27]. The criteria for selecting the pseudo-miRNAs from the segments are: minimum of 18 base pairings on the stem of the hairpin structure (included the GU wobble pairs), maximum of -15 kcal/mol free energy of the secondary structure, and no multiple loops. These criteria ensure that the extracted pseudo pre-miRNAs are similar to real pre-miRNAs according to the widely accepted characteristics. (The thresholds 18 and -15 are the lowest number of base pairings and the highest free energy among all the genuine human pre-miRNAs, respectively.) As all reported miRNAs are located in the un-translated regions or intergenic regions, we take the hairpins collected from CDS as examples of pseudo pre-miRNAs. Totally, 8,494 pre-miRNA-like hairpins are collected in this dataset.

The CONSERVED-HAIRPIN dataset is extracted from the genome region of position 56,000,001 to 57,000,000 on human chromosome 19. The data are obtained from the UCSC database (hg17, May 2004) [25]. The 659 sequences conserved between human and mouse in this region are collected, which contain 313,212 nucleotides. We use a window of width 100 nt to scan the region with step length 10 nt to produce sequence segments, the secondary structures of which are then predicted by RNAfold [27]. This results in 2,444 hairpins to compose the CONSERVED-HAIRPIN dataset according to the same criteria used for the CODING dataset. It should be noted that, unlike the CODING set, there might be a few true miRNAs among these segments. But since miRNAs only takes a very small proportion in the genome, most of the hairpins in this dataset are more likely pseudo-miRNAs. In fact, there are 3 known miRNAs (hsa-mir-99b, hsa-let-7e and hsa-mir-125a) in this dataset.

The CODING dataset is used as negative samples in the training and validation of the SVM classifier, and the CONSERVED-HAIRPIN dataset is used as a candidate dataset to evaluate how the classifier works on the genome.

### Training and test sets for classification experiments

For the classification experiments, one training set and two test sets are built using the datasets described above. The training set TR-C includes 163 human pre-miRNAs (positive samples) and 168 pseudo pre-miRNAs (negative samples) randomly selected from the 193 human pre-miRNAs and the CODING dataset, respectively. The test set TE-C comprises of the remaining 30 human pre-miRNAs not used in TR-C and 1000 pseudo pre-miRNAs randomly picked up from the CODING dataset (examples already selected in the training sets are avoided). The CONSERVED-HAIRPIN dataset is the second test set.

### CROSS-SPECIES test set

After experimenting on the human data, we apply the SVM classifier trained with human data to other species to see if the features are conserved during evolution. The release 5.0 of the miRNA registry [22,23] contains 1138 pre-miRNAs entries from 11 species besides human: *Caenorhabditis elegans*, *Caenorhabditis briggsae*, *Drosophila melanogaster*, *Drosophila pseudoobscura*, *Dnio rerio*, *Gallus gallus*, *Mus musculusi*, *Rattus norvegicus*, *Arabidopsis thaliana*, *Oryza sativa *and *Epstein Barr Virus*. Only the pre-miRNAs with no multiple loops are used, which cover more than 90% of all the reported pre-miRNAs of the 11 species. The pre-miRNAs that share high sequence similarities with the human pre-miRNAs are excluded to avoid biased evaluation of the SVM trained on human data. The similarity is calculated using BLASTCLUST [28] with S = 80, L = 0.5, W = 16. With these processing, 581 pre-miRNAs from the 11 species remained for the test experiment. We refer this set of pre-miRNAs as the CROSS-SPECIES test set.

### Latest human miRNA updated set

At the time this paper was being written, a batch of new human miRNAs are reported, most of which are not conserved beyond primates [14]. We took these latest data as an independent test set and applied our SVM classifier on it. From the 89 pre-miRNAs reported in [14], we extract the 88 that have no multiple loops. The sequence similarities between the 88 pre-miRNAs are calculated using BLASTCLUST with S = 80, L = 0.5, W = 16. Only one pre-miRNA is then chosen for each cluster to eliminate closely related sequences, which gives us 40 non-redundant pre-miRNAs. We also check the sequence similarity of these 40 pre-miRNAs with the 163 human pre-miRNAs in the training dataset and eliminate one more miRNA that has high similarity with entries in the training data. Finally, the remaining 39 pre-miRNAs are used as the UPDATED test set.

### The local contiguous structure-sequence features

Recent reports have shown that local sequence features are important in pre-miRNAs [29]. Our investigations show that the distributions of local contiguous sub-structures (continuously paired or unpaired structures) of pre-miRNAs are significantly distinct with that of pseudo pre-miRNAs. Based on these observations, we propose a set of features that combines the local contiguous structures with sequence information to characterize the hairpin structure of real vs. pseudo pre-miRNAs. The features focus on the information of every 3 adjacent nucleotides, and we name them as triplet structure-sequence elements or triplet elements for the convenience of discussion.

**Figure 1:**
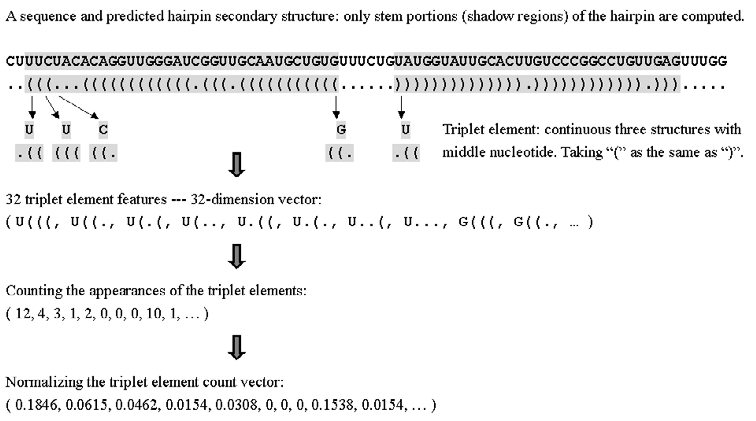
Using the triplet elements to represent the local structure-sequence features of the hairpin. The triplet element is composed of the 3 continuous sub-structures and the nucleotide type at the middle. The appearances of all 32 possible triplet elements are counted along a hairpin segment, forming a 32-dimensional vector, which is then normalized to be the input vector for SVM.

The RNA secondary structures are predicted using RNAfold [27]. In the predicted secondary structure, there are only two statuses for each nucleotide, paired or unpaired, indicated by brackets ("("or")") and dots ("."), respectively. The left bracket "(" means that the paired nucleotide is located near the 5'-end and can be paired with another nucleotide at the 3'-end, which is indicated by a right bracket")". We don't distinguish these two situations in this work and use "(" for both situations. For any 3 adjacent nucleotides, there are 8 (2^3^) possible structure compositions: "(((", "((.", "(..", "(.(", ".((", ".(.", "..(" and "...". Considering the middle nucleotide among the 3, there are 32 (4 × 8) possible structure-sequence combinations, which we denote as "U(((", "A((.", etc. This defines our triplet structure-sequence elements. As an example, Figure 1 illustrates how a hairpin is represented using triplet elements. We exclude the terminal loop and external single-stranded regions of the hairpin and only consider the stem portions. The number of appearance of each triplet element is counted for each hairpin (pre-miRNA or pseudo pre-miRNA) to produce the 32-dimensional feature vector. It is normalized before being used as input features for SVM.

### SVM classification

Support vector machine or SVM is used to classify real vs. pseudo pre-miRNAs with the 32-dimentional feature vectors. SVM is adopted due to its reported good generalization ability [30,31]. The SVM classifier is trained with the set TR-C and then applied on all the test sets. We call the whole strategy of using SVM with the triplet element features to recognize pre-miRNAs as the triplet-SVM method.

### Classification of human real vs. pseudo miRNA precursors

When applying SVM classifier on the set TE-C, 28 out of the 30 human pre-miRNAs are correctly recognized (the missed ones are "hsa-mir-147" and " hsa-mir-187") and 881 out of the 1000 pseudo-miRNAs are detected as negative, which gives a sensitivity of 93.3% and specificity of 88.1% (Table 1).

**Table 1:** Classification performance of the triplet-SVM classifier on test sets TE-C, CONSERVED-HAIRPIN and UPDATED.

Test set	Type	Size	Accuracy (%)
TE-C	Real^1^	30	93.3
	Pseudo^2^	1000	88.1
CONSERVED-HAIRPIN	Pseudo^2^	2444	89.0
UPDATED	Real^1^	39	92.3

^1^Real: real human pre-miRNAs.

^2^Pseudo: pseudo pre-miRNA hairpins.

On the set CONSERVED-HAIRPIN, the SVM classifier identifies 2,174 out of the 2,444 potential hairpin structures as false miRNAs, which give a specificity of or greater than 89.0% if we assume all the hairpins in this dataset are not true pre-miRNAs (but in fact there are at least 3 true pre-miRNAs and they are all correctly detected).

**Figure 2:**
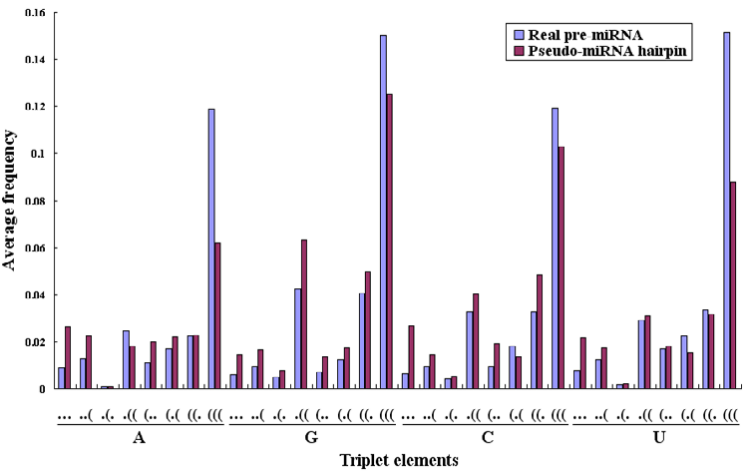
The average appearance frequencies of the triplet elements in the two classes (real pre-miRNA vs. pseudo-miRNA hairpins).

The high classification accuracies illustrate that the real and pseudo pre-miRNAs are quite distinct with regard to the triplet element features, though they share similar hairpin structure. The triplet elements reflect information of the local contiguous fine-structures and the sequence composition. For example, the triplet unit "(((" represents the stacking of paired bases and the unit "..." represents the interior or bugle loops, etc. The success of using these features to recognize real pre-miRNAs from other hairpins shows that these features might reflect some intrinsic characteristic of pre-miRNAs. We calculate the average occurring frequencies of the 32 triplet elements in the 163 pre-miRNAs and the 168 pseudo-miRNA hairpins in training dataset. Figure 2 displays the distribution of the average frequencies of triplet elements in the two classes. Comparing each of the basic structure units in the triplet elements between the real and pseudo pre-miRNAs, we can find that continuously paired nucleotides like "(((" appear at higher frequencies in pre-miRNAs than in pseudo-miRNAs, and continuously unpaired structures like "..." or "..(" tend to appear more often in pseudo-miRNAs.

A feature selection criterion reported in Dror *et al *[32] is adopted to analyze the discriminative powers of the different triplet elements. Denote *x*_*i*_, *i *= 1 ...32 as the 32 triplet element features. The means μi+
 MathType@MTEF@5@5@+=feaafiart1ev1aaatCvAUfKttLearuWrP9MDH5MBPbIqV92AaeXatLxBI9gBaebbnrfifHhDYfgasaacH8akY=wiFfYdH8Gipec8Eeeu0xXdbba9frFj0=OqFfea0dXdd9vqai=hGuQ8kuc9pgc9s8qqaq=dirpe0xb9q8qiLsFr0=vr0=vr0dc8meaabaqaciGacaGaaeqabaqabeGadaaakeaacqaH8oqBdaqhaaWcbaGaemyAaKgabaGaey4kaScaaaaa@30CE@, μi−
 MathType@MTEF@5@5@+=feaafiart1ev1aaatCvAUfKttLearuWrP9MDH5MBPbIqV92AaeXatLxBI9gBaebbnrfifHhDYfgasaacH8akY=wiFfYdH8Gipec8Eeeu0xXdbba9frFj0=OqFfea0dXdd9vqai=hGuQ8kuc9pgc9s8qqaq=dirpe0xb9q8qiLsFr0=vr0=vr0dc8meaabaqaciGacaGaaeqabaqabeGadaaakeaacqaH8oqBdaqhaaWcbaGaemyAaKgabaGaeyOeI0caaaaa@30D9@ and standard deviations σi+
 MathType@MTEF@5@5@+=feaafiart1ev1aaatCvAUfKttLearuWrP9MDH5MBPbIqV92AaeXatLxBI9gBaebbnrfifHhDYfgasaacH8akY=wiFfYdH8Gipec8Eeeu0xXdbba9frFj0=OqFfea0dXdd9vqai=hGuQ8kuc9pgc9s8qqaq=dirpe0xb9q8qiLsFr0=vr0=vr0dc8meaabaqaciGacaGaaeqabaqabeGadaaakeaacqaHdpWCdaqhaaWcbaGaemyAaKgabaGaey4kaScaaaaa@30DB@, σi−
 MathType@MTEF@5@5@+=feaafiart1ev1aaatCvAUfKttLearuWrP9MDH5MBPbIqV92AaeXatLxBI9gBaebbnrfifHhDYfgasaacH8akY=wiFfYdH8Gipec8Eeeu0xXdbba9frFj0=OqFfea0dXdd9vqai=hGuQ8kuc9pgc9s8qqaq=dirpe0xb9q8qiLsFr0=vr0=vr0dc8meaabaqaciGacaGaaeqabaqabeGadaaakeaacqaHdpWCdaqhaaWcbaGaemyAaKgabaGaeyOeI0caaaaa@30E6@ of *x*_*i *_in the two classes are calculated. The discriminatory power of each triplet element is assessed by an *F *value defined as F(xi)=|μi+−μi−σi++σi−|
 MathType@MTEF@5@5@+=feaafiart1ev1aaatCvAUfKttLearuWrP9MDH5MBPbIqV92AaeXatLxBI9gBaebbnrfifHhDYfgasaacH8akY=wiFfYdH8Gipec8Eeeu0xXdbba9frFj0=OqFfea0dXdd9vqai=hGuQ8kuc9pgc9s8qqaq=dirpe0xb9q8qiLsFr0=vr0=vr0dc8meaabaqaciGacaGaaeqabaqabeGadaaakeaacqWGgbGrcqGGOaakcqWG4baEdaWgaaWcbaGaemyAaKgabeaakiabcMcaPiabg2da9maaemaabaWaaSaaaeaacqaH8oqBdaqhaaWcbaGaemyAaKgabaGaey4kaScaaOGaeyOeI0IaeqiVd02aa0baaSqaaiabdMgaPbqaaiabgkHiTaaaaOqaaiabeo8aZnaaDaaaleaacqWGPbqAaeaacqGHRaWkaaGccqGHRaWkcqaHdpWCdaqhaaWcbaGaemyAaKgabaGaeyOeI0caaaaaaOGaay5bSlaawIa7aaaa@495E@. Table 2 lists the 15 most discriminative triplet elements. It can be seen that the most informative triplet elements are the continuously paired or unpaired structures. It should be emphasized that the pseudo-miRNAs collected in this study are also "good" hairpins selected with a series of strict criteria according to the existing understanding of pre-miRNA hairpins. Our observations show that the major differences between pre-miRNAs and other similar hairpins are in these kinds of fine structure features. This can be related with the stability of the secondary structure of the pre-miRNAs. Essentially, the stacking of more continuously paired nucleotides can decrease the free energy of the folded structure and stabilize of the secondary structure, whereas the occurring of interior loops and bugle loops (like the "..." and "..(" in the triplet elements) can destabilize the RNA structures by increasing the free energy [33]. So, these results manifest that pre-miRNAs are more stable than other hairpins with similar stem-loop structures in the genome. It has been suggested that the stability of pre-miRNA might be associated with the biogenesis and processing procedure of mature miRNAs [28].

**Table 2:** The discriminative power of top 15 triplet elements. The discriminative power of the triplet element features that distinguish pre-miRNAs from other similar hairpins are calculated using the *F *value and the 15 most discriminative triplet elements are listed here. The *μ*^+^, *μ*^- ^and *σ*^+^, *σ*^- ^are the means and standard deviations of the elements in the two classes estimated with the training dataset

Triplet elements	Pre-miRNAs		Other hairpins		
			
	*μ*^+^	*σ*^+^	*μ*^-^	*σ*^-^	*F*
**A(((**	0.121	0.042	0.063	0.032	0.792
**U(((**	0.154	0.048	0.089	0.040	0.734
**C...**	0.006	0.011	0.025	0.030	0.475
**A...**	0.008	0.014	0.025	0.025	0.429
**U...**	0.007	0.011	0.021	0.023	0.397
**G.((**	0.042	0.025	0.063	0.031	0.383
**C(..**	0.009	0.011	0.019	0.017	0.353
**C((.**	0.032	0.022	0.048	0.027	0.329
**A(..**	0.011	0.012	0.020	0.016	0.316
**G(((**	0.151	0.038	0.127	0.040	0.303
**A..(**	0.013	0.013	0.022	0.019	0.295
**G...**	0.006	0.011	0.014	0.019	0.289
**G(..**	0.007	0.010	0.014	0.015	0.266
**G((.**	0.040	0.020	0.050	0.024	0.231
**C(((**	0.119	0.030	0.105	0.034	0.230

Sequence information is also included in the triplet elements. Previous studies of pre-miRNAs have carefully considered the effect of primary sequence orders. Distributions of mono-nucleotides and di-nucleotides are often preserved when producing comparable sequences [29]. Our experiments show that the appearance frequencies of the same triplet structure units with different middle nucleotides in real pre-miRNAs are not identical, and their appearance frequencies between real and pseudo miRNAs are significantly distinct (see Figure 2). Experiments also show that SVM performs better when taking the sequence information into the triplet elements, than using just the 8 triplet structure features without the nucleotide information. For example, we trained another SVM classifier with same training dataset using only the 8 triplet structure features. When being applied to the set TE-C, the SVM classifier correctly recognized 29 out of 30 human pre-miRNAs (sensitivity 96.7%), but it only detected 636 pseudo-miRNAs as negative (specificity 63.6%). On the CONSERVED-HAIRPIN set, that SVM classifier identified 1702 out of the 2444 potential hairpin structures as false pre-miRNAs, which gave specificity of or above 69.6%. We can see that the specificity can be greatly improved when using both structure and sequence information.

### Applying to all other species

Most, if not all, existing miRNA prediction methods work on animals and plants separately, since plant miRNAs are known to have more heterogeneous hairpin structures than animal miRNAs [2,34]. Interestingly, when we apply the SVM classifier trained with only human data to all other species (ranging from animals, plants and virus) where miRNAs have been reported, it can correctly identify most of the true pre-miRNAs. Table 3 shows the SVM prediction on the CROSS-SPECIES set, which contains 581 known pre-miRNAs of 11 species. Any pre-miRNAs that are homologous to the human miRNAs have been excluded from the set. The SVM classifier achieves an overall accuracy of 90.9% on the CROSS-SPECIES set. Especially, it is noticeable that the classifier correctly identifies most the plant and viral pre-miRNAs. It is known that plant miRNAs usually have longer precursor sequences [29]. The success of the triplet-SVM classifier across the wide range of different species indicates that there may be local contiguous structure-sequence characteristics that are conserved in pre-miRNAs of all species. Similarly, when comparing the appearance frequencies of each triplet elements in real pre-miRNAs within all the species vs. the pseudo pre-miRNAs, their differences are well conserved.

**Table 3:** Prediction accuracy on test set CROSS-SPECIES by SVM trained with human data.

Species	# of pre-miRNAs	Accuracy (%)
*Mus musculusi*	36	94.4
*Rattus norvegicus*	25	80
*Callus gallus*	13	84.6
*Dnio rerio*	6	66.7
*Caenorhabditis briggsae*	73	95.9
*Caenorhabditis elegans*	110	86.4
*Drosophila pseudoobscura*	71	90.1
*Drosophila melanogaster*	71	91.5
*Oryza sativa*	96	94.8
*Arabidopsis thaliana*	75	92
*Epstein Barr Virus*	5	100
Total	581	90.9

### Testing on the latest human-specific miRNAs

The 39 human pre-miRNAs from in the UPDATED set were newly reported when this work was almost completed. They are not conserved in closely related species, and therefore existing homology-based methods all fail to identify them [14,35]. With our triplet-SVM classifier, 36 of the 39 new pre-miRNAs are correctly recognized, giving an accuracy of 92.3% (Table 1). This shows that *ab initio *miRNA predictors like the proposed triplet-SVM method can be more powerful in discovering novel or species-specific pre-miRNAs.

## Conclusion

A major characteristic that defines miRNA precursors is the hairpin structures, but large amounts of similar hairpins can be formed from sequence segments in genomes. *Ab initio *method for distinguishing true pre-miRNAs from other pre-miRNA-like hairpin structures is important for discovering new and species-specific miRNAs. For this purpose, a set of novel features (the triplet elements) to describe local contiguous structure-sequence characteristics are extracted, and support vector machine is applied with these features to classify real vs. pseudo pre-miRNAs, achieving about 90% accuracy on human test data. Remarkably, the triplet-SVM classifier built on human data can correctly classify up to 90% of the pre-miRNAs from the other 11 species including plants and virus without utilizing any comparative genomics information and an accuracy of 92.3% is achieved on the newly reported novel human miRNAs. The local structure-sequence features may contain distinctive and conserved characteristics of miRNAs, and the successful *ab initio *classification of real and pseudo pre-miRNAs opens a new approach for discovering new miRNAs.

Scanning the genome, there could be numerous amounts of sequence segments that can be folded into pre-miRNA-like hairpins. The ability to distinguish pseudo vs. real pre-miRNAs is essential in the computational identification of novel and species-specific miRNAs. Since the number of possible candidate hairpins is very large, the current specificity around 89% is still not satisfactory for genome-wide applications and a lot of false positive predictions can be produced. How to find more information to further reduce the false positive rate is what should be sought next. However, latest reports suggested that there may be much more miRNAs than the number currently known [35]. It might be necessary to reconsider what we previously regard as false-positive predictions. The successful application of the human-based classifier on all other species implies that the biogenesis and processing mechanism of miRNAs might be conserved between animals, plants and viruses.

## Methods

### Support vector machine

The basic principle of SVM is: For a given data set *x*_*i *_∊ *R*^*n *^(*i *= 1,... *N*) with corresponding labels *y*_*i *_(*y*_*i *_= +1 or -1, representing the two classes to be classified, as real pre-miRNA vs. pseudo pre-miRNA in this study), SVM gives a decision function (classifier) f(x)=sgn⁡(∑i=1NyiαiK(x,xi)+b)
 MathType@MTEF@5@5@+=feaafiart1ev1aaatCvAUfKttLearuWrP9MDH5MBPbIqV92AaeXatLxBI9gBaebbnrfifHhDYfgasaacH8akY=wiFfYdH8Gipec8Eeeu0xXdbba9frFj0=OqFfea0dXdd9vqai=hGuQ8kuc9pgc9s8qqaq=dirpe0xb9q8qiLsFr0=vr0=vr0dc8meaabaqaciGacaGaaeqabaqabeGadaaakeaacqWGMbGzcqGGOaakcqWG4baEcqGGPaqkcqGH9aqpcyGGZbWCcqGGNbWzcqGGUbGBcqGGOaakdaaeWbqaaiabdMha5naaBaaaleaacqWGPbqAaeqaaOGaeqySde2aaSbaaSqaaiabdMgaPbqabaGccqWGlbWscqGGOaakcqWG4baEcqGGSaalcqWG4baEdaWgaaWcbaGaemyAaKgabeaakiabcMcaPiabgUcaRiabdkgaIjabcMcaPaWcbaGaemyAaKMaeyypa0JaeGymaedabaGaemOta4eaniabggHiLdaaaa@4F67@, where *α*_*i *_are the coefficients to be learned and *K *is a kernel function. Parameters *α*_*i *_'s are trained through maximizing ∑i=1Nαi−12∑i,j=1NαiαjyiyjK(xi,xj),subject to0≤αi≤C (i=1,…N)and∑i−1Nαiyi=0
 MathType@MTEF@5@5@+=feaafiart1ev1aaatCvAUfKttLearuWrP9MDH5MBPbIqV92AaeXatLxBI9gBaebbnrfifHhDYfgasaacH8akY=wiFfYdH8Gipec8Eeeu0xXdbba9frFj0=OqFfea0dXdd9vqai=hGuQ8kuc9pgc9s8qqaq=dirpe0xb9q8qiLsFr0=vr0=vr0dc8meaabaqaciGacaGaaeqabaqabeGadaaakeaafaqabeqafaaaaeaadaaeWbqaaiabeg7aHnaaBaaaleaacqWGPbqAaeqaaOGaeyOeI0YaaSaaaeaacqaIXaqmaeaacqaIYaGmaaWaaabCaeaacqaHXoqydaWgaaWcbaGaemyAaKgabeaakiabeg7aHnaaBaaaleaacqWGQbGAaeqaaOGaemyEaK3aaSbaaSqaaiabdMgaPbqabaGccqWG5bqEdaWgaaWcbaGaemOAaOgabeaakiabdUealjabcIcaOiabdIha4naaBaaaleaacqWGPbqAaeqaaOGaeiilaWIaemiEaG3aaSbaaSqaaiabdQgaQbqabaGccqGGPaqkcqGGSaalaSqaaiabdMgaPjabcYcaSiabdQgaQjabg2da9iabigdaXaqaaiabd6eaobqdcqGHris5aaWcbaGaemyAaKMaeyypa0JaeGymaedabaGaemOta4eaniabggHiLdaakeaaieaacqWFZbWCcqWF1bqDcqWFIbGycqWFQbGAcqWFLbqzcqWFJbWycqWF0baDcaaMc8Uae8hDaqNae83Ba8gabaGaeGimaaJaeyizImQaeqySde2aaSbaaSqaaiabdMgaPbqabaGccqGHKjYOcqWGdbWqcaaMc8UaeiikaGIaemyAaKMaeyypa0JaeGymaeJaeiilaWIaeSOjGSKaemOta4KaeiykaKcabaGae8xyaeMae8NBa4Mae8hzaqgabaWaaabCaeaacqaHXoqydaWgaaWcbaGaemyAaKgabeaakiabdMha5naaBaaaleaacqWGPbqAaeqaaOGaeyypa0JaeGimaadaleaacqWGPbqAcqGHsislcqaIXaqmaeaacqWGobGta0GaeyyeIuoaaaaaaa@8CA1@.

The LibSVM package (version 2.36) [36] is used. To obtain SVM classifier with optimal performance, the penalty parameter C and the RBF kernel parameter *γ *are tuned based on the training set using the grid search strategy in LibSVM.

### Software availability

The program of the presented triplet-SVM classifier can be freely accessible on our website at [37]. More detailed data of the experiments are also provided at the site.

## Authors' contributions

CX and FL developed the methods and CX wrote the codes and implemented most of the experiments under the guide of XZ. TH assisted in collecting and pre-processing the data and provided helpful insight in the method development. GPL helped the writing. YL and XZ initiated the project and guided the forming of the ideas. CX and XZ did most of the writing with inputs from all authors.
